# Online Medical Record Nonuse Among Patients: Data Analysis Study of the 2019 Health Information National Trends Survey

**DOI:** 10.2196/24767

**Published:** 2021-02-22

**Authors:** Safa Elkefi, Zhongyuan Yu, Onur Asan

**Affiliations:** 1 School of Systems and Enterprises Stevens Institute of Technology Hoboken, NJ United States

**Keywords:** online medical records, cancer, patient portals, communication, medical records, health information, eHealth

## Abstract

**Background:**

Online medical records are being used to organize processes in clinical and outpatient settings and to forge doctor-patient communication techniques that build mutual understanding and trust.

**Objective:**

We aimed to understand the reasons why patients tend to avoid using online medical records and to compare the perceptions that patients have of online medical records based on demographics and cancer diagnosis.

**Methods:**

We used data from the Health Information National Trends Survey Cycle 3, a nationally representative survey, and assessed outcomes using descriptive statistics and chi-square tests. The patients (N=4328) included in the analysis had experienced an outpatient visit within the previous 12 months and had answered the online behavior question regarding their use of online medical records.

**Results:**

Patients who were nonusers of online medical records consisted of 58.36% of the sample (2526/4328). The highest nonuser rates were for patients who were Hispanic (460/683, 67.35%), patients who were non-Hispanic Black (434/653, 66.46%), and patients who were older than 65 years (968/1520, 63.6%). Patients older than 65 years were less likely to use online medical records (odds ratio [OR] 1.51, 95% CI 1.24-1.84, *P*<.001). Patients who were White were more likely to use online medical records than patients who were Black (OR 1.71, 95% CI 1.43-2.05, *P*<.001) or Hispanic (OR 1.65, 95% CI 1.37-1.98, *P*<.001). Patients who were diagnosed with cancer were more likely to use online medical records compared to patients with no cancer (OR 1.31, 95% CI 1.11-1.55, 95% CI 1.11-1.55, *P*=.001). Among nonusers, older patients (≥65 years old) preferred speaking directly to their health care providers (OR 1.76, 95% CI 1.35-2.31, *P*<.001), were more concerned about privacy issues caused by online medical records (OR 1.79, 95% CI 1.22-2.66, *P*<.001), and felt uncomfortable using the online medical record systems (OR 10.55, 95% CI 6.06-19.89, *P*<.001) compared to those aged 18-34 years. Patients who were Black or Hispanic were more concerned about privacy issues (OR 1.42, 1.09-1.84, *P*=.007).

**Conclusions:**

Studies should consider social factors such as gender, race/ethnicity, and age when monitoring trends in eHealth use to ensure that eHealth use does not induce greater health status and health care disparities between people with different backgrounds and demographic characteristics.

## Introduction

Communication is a key element in providing high-quality health care services [1]. If communication is effective, it can lead to significant health care provision and positive outcomes, including decreased anxiety, guilt, pain, and disease symptoms. Moreover, effective communication can increase patient satisfaction, acceptance, adherence, and cooperation with the medical team, and it can improve the physiological and functional status of the patient [1]. Conversely, poor communication between doctors and patients can lead to poor quality and continuity of care [2]. Patients with communication-related disabilities or problems were 3 times more likely to experience medical or clinical complications compared to other patients [3]. Thus, ensuring good communication by recording, handling, and sharing health information with patients remains a necessity and an integral component of the health care process that may improve outcomes and limit or prevent costly duplication of tests and treatments [4,5]. 

Information exchange is not only limited to the visit. It is moving beyond that, as patients have the opportunity to access their information regardless of location or time via technologies [4]. Technological advancement has provided opportunities not only to improve doctor-patient information exchange but also to inform and empower patients’ role in decision making. Information and communication technology is often used for communication and information exchange purposes and may be a valuable tool for handling these challenges [6,7]. Other potential solutions include means for patients to access their online medical records, including personalized health records [8-11] and patient portals [12]. These centralized health information systems could also have the greatest benefit for information transmission. Although patient portals such as MyPreventiveCare [13], Ask an Expert [14], and others are considered important tools for the development of patient-centered care, their current use is not optimal, and portals are still less patient-centered than they could be [15-17]. Known barriers to the use of portals for patients and providers include security and privacy concerns, the potential negative impact on provider workflow, and limited user friendliness [12,18].

The ability of patients or individuals to access their online medical records serves as one of the backbones to improve patient engagement in and the outcomes of our health care system. Historically, patients have had low access to online medical records. For instance, only 3 out of 10 patients were offered access to medical records in 2013, and almost half of those offered access viewed their online records at least once [19]. However, the use of online medical records has experienced significant growth in recent years, and technological improvements have been made to improve usability and implementation [20]. On the other hand, despite greater availability, there are many patients who still avoid using online medical records, though this may be changing with the current COVID-19 pandemic paradigm shift. The purpose of this study was to explore factors leading to the use or nonuse of online medical records across different groups of patients.

## Methods

### Data Source

Data for this study were derived from the National Cancer Institute’s 2019 Health Information National Trends Survey (HINTS). HINTS is a nationally representative survey (of the US noninstitutionalized adult population) that collects data on the American public’s need for, access to, and use of health-related information [21]. HINTS is publicly available on the web [22]. Data used in this study were from HINTS 5 Cycle 3, collected between January and May of 2019. Patients who gave information about their online medical records use were included (survey response rate: 4328/14332, 30.20%). Further details on survey design and sampling strategies are published elsewhere [23].

### Study Variables

The following questions correspond to the measures used in the analysis of the study. The first question was, “How many times did you access your online medical record in the last 12 months?” We used this question to identify users and nonusers of online medical records. The respondents who reported accessing their medical record at least once were coded as *users* and respondents who reported accessing their records zero times were coded as *nonusers*. The primary population of interest of the study was nonusers. We specifically analyzed the responses of nonusers to the following questions (Table 1) regarding the reasons why they do not use online medical records.

**Table 1:** Questions for nonusers and corresponding factors.

Factor name	Question^a^
SpeakDirectly	You prefer to speak to your health care provider directly?
NoNeed	You did not have a need to use your online medical record?
ConcernedPrivacy	You were concerned about the privacy or security of your medical records’ website?
NoRecord	You do not have an online medical record?
LogInProb	You found it difficult to login (for example, you had trouble remembering your password)?
Uncomfortable	You are not comfortable or experienced with computers?
MultipleRec	You have more than one online medical record?

^a^All questions had binary (yes or no) responses.

### Data Analysis

Descriptive statistics for the HINTS 5 Cycle 3 populations were generated for demographic variables (gender, age, race/ethnicity) and the cancer diagnosis variable. We investigated the relationship between the use of online medical records and the demographic or diagnosis variables using an unadjusted model—Fisher exact test. We focused on patients who had not used online medical records to better understand the reasons behind their avoidance of this particular technology. We report odds ratios (OR) and corresponding 95% confidence intervals; statistical significance was determined based on *P*<.05. All analyses were conducted using R statistical software (version 3.6.3; packages: Ime4 and Stats).

## Results

### Sample Characteristics

HINTS 5 Cycle 3 had a total of 4328 respondents, and 57.74% of the respondents (2499/4328) were female. Most respondents were older than 50 years (2926/4328, 67.61%) and non-Hispanic White (2992/4328, 69.13%), and 16% of respondents (693/4328) had been diagnosed with cancer (Table 2).

**Table 2:** Demographic characteristics of the patients.

Characteristic		Respondents (N=4328), n (%)	
**Gender**			
	Male		1829 (42.26)
	Female		2499 (57.74)
**Age (years)**			
	18-34		580 (13.40)
	35-49		822 (18.99)
	50-64		1406 (32.49)
	≥65		1520 (35.12)
**Race**			
	Hispanic		683 (15.78)
	Non-Hispanic White		2992 (69.13)
	Non-Hispanic Black		653 (15.09)
**Cancer diagnosis**			
	Have cancer		693 (16.01)
	No cancer		3635 (83.99)

### Use of Online Medical Records

Among the 4328 respondents, 2526 (58.36%) were nonusers. The ratio of nonusers across demographics ranged between 53% to 67%, with patients who were Hispanic (460/683, 67.35%) and patients who were non-Hispanic Black being highest (434/653, 66.46%). Patients who were non-Hispanic White (1360/2992, 45.45%) and patients between 18 and 34 years of age had the highest online medical record use (269/580, 46.38%). Table 3 presents percentages of users and nonusers across demographics and nonuser comparisons.

**Table 3:** Use of online medical records.

Characteristics		All, n (%)	Nonuser, n (%)	User, n (%)	Odds ratio (95% CI)	*P* value
All		4328	2526 (58.36)	1802 (41.64)	N/A^a^	N/A
**Gender**						
	Male	1829 (42.26)	1130 (61.78)	699 (38.22)	1	N/A
	Female	2499 (57.74)	1396 (55.86)	1103 (44.14)	0.78 (0.69-0.88)	*<*.001
**Age (years)**						
	18-34	580 (13.40)	311 (53.62)	269 (46.38)	1	N/A
	35-49	822 (18.99)	450 (54.74)	372 (45.26)	1.04 (0.84-1.30)	
	50-64	1406 (32.49)	797 (56.69)	609 (43.31)	1.13 (0.92-1.38)	
	≥65	1520 (35.12)	968 (63.68)	552 (36.32)	1.51 (1.24-1.84)	*<*.001
**Race**						
	Non-Hispanic White	2992 (69.13)	1632 (54.55)	1360 (45.45)	1	N/A
	Hispanic	683 (15.78)	460 (67.35)	223 (32.65)	1.71 (1.43-2.05)	*<*.001
	Non-Hispanic Black	653 (15.09)	434 (66.46)	219 (33.54)	1.65 (1.37-1.98)	*<*.001
**Cancer diagnosis**						
	Have cancer	693 (16.01)	365 (52.67)	328 (47.33)	1	N/A
	No cancer	3635 (83.99)	2161 (59.95)	1474 (40.05)	1.31 (1.11-1.55)	*<*.001

^a^N/A: not applicable.

Patients who avoided using online medical records were more likely to be male (OR 0.78, 95% CI 0.69-0.88, *P<*.001). The oldest patients (aged >65 years) were less likely to use online medical records (OR 1.51, 95% CI 1.24-1.84, *P<*.001). Patients who were non-Hispanic White were more likely to use online medical records than patients who were Hispanic (OR 1.71, 95% CI 1.43-2.05, *P<*.001) or non-Hispanic Black (OR 1.65, 95% CI 1.37-1.98, *P<*.001). Finally, patients diagnosed with cancer were more likely to use online medical records than patients without cancer (OR 1.31, 95% CI 1.11-1.55, *P*=.001).

### Reasons for Nonuse of Online Medical Records

In the second phase of the analysis, we only focused on nonusers (n=2526) and explored factors regarding their preference for not using online medical records. We compared the different demographics for each factor. The survey had 7 listed factors asked to each respondent. Each participant responded to each question with “yes” or “no.” We summarized the percentage for each demographic group among nonusers. The “desire to speaking directly to the health care provider” was the primary factor (1575/2526, 62.35%) influencing the nonuse of online medical records. Across all demographic characteristics, more than half of respondents answered “yes” for this question. For instance, 63.68% (889/1396) of female nonusers reported “desire to speaking directly” as one of the primary reasons. Almost half of the participants also expressed “no need” as a reason to avoid online medical records. The 18-34 years age group of nonusers had the highest rate of “no need” factor to explain their avoidance of online medical record use (194/311, 62.38%). Privacy concerns were not a primary reason to avoid online medical record use across all groups (range 12% to 23%). Table 4 illustrates all descriptive statistics for the overall nonuser population and each demographic group.

**Table 4:** Frequencies of reasons that explain nonuse of online medical records.

Characteristic		Factor, n (%)						
		SpeakDirectly	NoNeed	ConcernedPrivacy	NoRecord	LogInProb	Uncomfortable	MultipleRec
**All**		1575 (62.35)	1258 (49.80)	499 (19.75)	571 (22.60)	435 (17.22)	547 (21.65)	228 (9.03)
**Gender**								
	Male	686 (60.71)	594 (52.57)	214 (18.94)	288 (25.49)	170 (15.04)	253 (22.39)	109 (9.65)
	Female	889 (63.68)	664 (47.56)	285 (20.42)	283 (20.27)	265 (18.98)	294 (21.06)	119 (8.52)
**Age (years)**								
	18-34	166 (53.38)	194 (62.38)	39 (12.54)	93 (29.90)	45 (14.47)	14 (4.50)	26 (8.36)
	35-49	231 (51.33)	252 (56.00)	83 (18.44)	114 (25.33)	71 (15.78)	48 (10.67)	35 (7.78)
	50-64	530 (66.50)	386(48.43)	179 (22.46)	173 (21.71)	130 (16.31)	163 (20.45)	77 (9.66)
	≥65	648 (66.94)	426 (44.01)	198 (20.45)	191 (19.73)	189 (19.52)	322 (33.26)	90 (9.30)
**Race**								
	Non-Hispanic White	1004 (61.52)	922 (56.50)	289 (17.71)	375 (22.98)	294 (18.01)	334 (20.47)	164 (10.05)
	Hispanic	294 (63.91)	172 (37.39)	108 (23.48)	100 (21.74)	91 (19.78)	118 (25.65)	37 (8.04)
	Non-Hispanic Black	277 (63.82)	164 (37.79)	102 (23.50)	96 (22.12)	50 (11.52)	95 (21.89)	27 (6.22)
**Cancer diagnosis**								
	Have cancer	260 (71.23)	190 (52.05)	74 (20.27)	76 (20.82)	79 (21.64)	117 (32.05)	50 (13.70)
	No cancer	1315 (60.85)	1068 (49.42)	425 (19.67)	495 (22.91)	356 (16.47)	430 (19.90)	178 (8.24)

The statistical analysis also yielded significant differences across the different demographics (Table 5). Older patients (≥65 years old) were more likely to avoid using online medical records. They preferred speaking directly to their health care providers (OR 1.76, 95% CI 1.35-2.31, *P<*.001), were more concerned about privacy issues caused by online medical records (OR 1.79, 95% CI 1.22-2.66, *P<*.001), and felt uncomfortable using the systems (OR 10.55, 95% CI 6.06-19.89, *P<*.001) compared to patients aged 18-34 years; however, they were more likely to be in need of online records (OR 0.47, 95% CI 0.36-0.62, *P<*.001).

**Table 5:** Reasons for not using online medical records.

Characteristic		Odds ratio (95% CI)						
		SpeakDirectly	NoNeed	ConcernedPrivacy	NoRecord	LogInProb	Uncomfortable	MultipleRec
**Gender**								
	Male	1.00 (1.00-1.00)	1.00 (1.00-1.00)	1.00 (1.00-1.00)	1.00 (1.00-1.00)	1.00 (1.00-1.00)	1.00 (1.00-1.00)	1.00 (1.00-1.00)
	Female	1.13 (0.96-1.33)	0.81 (0.69-0.96)*	1.09 (0.89-1.34)	0.74 (0.61-0.90)**	1.21 (0.96-1.54)	0.92 (0.76-1.12)	0.87 (0.65-1.15)
**Age (years)**								
	18-34	1.00 (1.00-1.00)	1.00 (1.00-1.00)	1.00 (1.00-1.00)	1.00 (1.00-1.00)	1.00 (1.00-1.00)	1.00 (1.00-1.00)	1.00 (1.00-1.00)
	35-49	0.92 (0.68-1.24)	0.76 (0.56-1.04)	1.57 (1.02-2.44)*	0.79 (0.56-1.11)	1.10 (0.72-1.70)	2.53 (1.34-5.06)**	0.92 (0.52-1.63)
	50-64	1.73 (1.31-2.2)***	0.56 (0.42-0.74)***	2.01 (1.37-3.01)	0.65 (0.47-0.88)**	1.15 (0.78-1.70)	5.44 (3.08-10.36)*	1.17 (0.72-1.94)
	>65	1.76 (1.35-2.31)***	0.47 (0.36-0.62)***	1.79 (1.22-2.66)**	0.57 (0.42-0.77)*	1.43 (0.99-2.09)	10.55 (6.06-19.89)***	1.12 (0.70-1.84)
**Race**								
	Non-Hispanic White	1.00 (1.00-1.00)	1.00 (1.00-1.00)	1.00 (1.00-1.00)	1.00 (1.00-1.00)	1.00 (1.00-1.00)	1.00 (1.00-1.00)	1.00 (1.00-1.00)
	Hispanic	1.10 (0.88-1.38)	0.46 (0.36-0.57)***	1.42 (1.09-1.84)	0.93 (0.71-1.20)	1.12 (0.85-1.46)	1.34 (1.04-1.71) *	0.78 (0.52-1.14)
	Non-Hispanic Black	1.10 (0.88-1.38)	0.46 (0.37-0.58)***	1.42 (1.09-1.84)	0.95 (0.72-1.23)	0.59 (0.42-0.82)**	1.08 (0.83-1.41)	0.59 (0.37-0.91)*
**Cancer**								
	Have cancer	1.00 (1.00-1.00)	1.00 (1.00-1.00)	1.00 (1.00-1.00)	1.00 (1.00-1.00)	1.00 (1.00-1.00)	1.00 (1.00-1.00)	1.00 (1.00-1.00)
	No cancer	0.62 (0.48-0.80)***	0.90 (0.71-1.13)	0.96 (0.72-1.28)	1.12 (0.85-1.50)	0.71 (0.53-0.95)*	0.52 (0.41-0.67)***	0.56 (0.40-0.80)**

*P<.05; **P<.01; ***P<.001.

Female respondents were more likely to use online medical records, and they were more likely to have records (OR 0.74, 95% CI 0.61-0.90, *P*=.003 for female). Patients who were Black or Hispanic were more concerned about privacy issues (Black: OR 1.42, 95% CI 1.09-1.84, *P*=.007; Hispanic: OR 1.42, 95% CI 1.09-1.84, *P*=.007) but were more conscious about the need for online records (Black: OR 0.46, 95% CI 0.37-0.58, *P<*.001; Hispanic: OR 0.46, 95% CI 0.36-0.57, *P<*.001). Patients who were Black were more likely to have problems logging in to their records (OR 0.59, 95% CI 0.42-0.82, *P*=.002).

Patients with cancer preferred to speak directly to the health care providers (OR 0.62, 95% CI 0.48-0.80, *P<*.001) and were more likely to feel uncomfortable using online medical records (OR 0.52, 95% CI 0.41-0.67, *P<*.001).

## Discussion

### General

Many studies that aim to improve information sharing and technology use in health care settings are based on exploring design improvements in patient-centered tools. Some discuss the environmental and technical barriers of adopting these tools [12,18], and others focus on providing training to enhance use-related skills [24]. We used nationally representative data from 2019 and examined attitude and other factors influencing nonuse of online medical records across different groups of patients. The results portray trends just prior to the pandemic, which has undoubtedly precipitated a paradigm shift toward online and telehealth medical use since the beginning of 2020.

The results showed that online medical record use was improving compared to use in previous years—41% overall in 2019 compared to 28% in 2017 [25]. However, online medical record use was still only approximately 30% for older adults (65 years and above) and respondents who were Black or Hispanic. Older adults were less likely to use online medical records compared to younger patients. Previous studies have also found that those older than 65 years would be less likely to use the internet to find health information [26,27] and less likely to use electronic personal health records [28]. Another study [29,30] also demonstrated that older adults have rather negative attitudes toward computers. Patients who were Black or Hispanic in our sample were more likely to be nonusers of online medical records than patients who were White. This was consistent with the findings of a previous study [27] that collected data between 2010 and 2017; however, in our study, there were increased rates of online medical record use among minorities compared to those of previous years. Due to chronic underlying health conditions, adults aged ≥65 years and individuals who are Black or Hispanic were the two groups hit hardest by the COVID-19 pandemic [31]. These groups often need continued care due to chronic health conditions [32]; however, visiting hospitals and clinics during the pandemic may increase the risk of infection. The online medical use rate would likely to be increased among these groups due to the pandemic.

In this study, we explicitly focused on the reasons people avoid online medical record use across demographics. The major reason that emerged from the data is that patients would prefer to “see their physicians in person.” This was the primary reason for all groups except younger patients. Older patients were more likely to prefer to “speak in person with physicians” than younger patients. Furthermore, the preference to “speak in person with physicians” was also high for all race groups (1575/2526, 62.35%)*.* These findings are not surprising since most patients value in-person visits with their doctors due to the more personal nature of the interaction, the opportunity to use nonverbal cues, and the ability to explain specific symptoms to doctors more clearly. Since March of 2020, however, the health care system is experiencing a paradigm shift due to an unprecedented pandemic, and telehealth visits have become a new normal, often replacing in-person visits, especially for older and chronically ill patients who are at high risk for COVID-19 infection. There is a tremendous rise in virtual care via telehealth technology during the pandemic [33]. Future studies will undoubtedly show changes in the demand for in-person visits after these forced experiences.

In the event of a population-wide infectious disease outbreak such as COVID-19, people’s online activities may affect public concerns and health behaviors. Many studies [34,35] have explored people’s active use of online information in various crises, including a public health crisis. The recent COVID-19 pandemic also sparked a paradigm shift in using online health care communication tools such as telehealth. It showed the importance and necessity of information sharing and communication beyond the walls of clinics.

The technology acceptance model explains that people use technologies when they are perceived as useful and necessary [36]. Almost half of the nonusers stated that there was no need for them to use online medical records. Young respondents (aged 18-34 years) among the sample were shown to be more likely to use online electronic medical records. Those who did not use online medical records in this group were likely to express that they had “no need” for their online medical record during this time period. This showed a significant difference compared to older patients (*P<*.001). Intuitively, younger patients are healthier than older patients, but they also tend to be more comfortable using online health technologies. After the COVID-19 pandemic, a new necessity may emerge which also might influence young patients use of online medical records for any information exchange with their providers. Future HINTS data collected during or after the pandemic might also show this shift among young patients. Finally, respondents who were Black or Hispanic were less likely to state the reason of “no need” than respondents who were White.

Privacy has always been an issue for some users regarding the use of technologies for information sharing, especially information as sensitive and personal as medical records. Some participants also declared this as one of the factors for avoiding online medical record use. Respondents who were Black or Hispanic were more likely to have privacy concerns compared to those who were White. Historically, minorities have less trust in the health care system due to disparities they have experienced [24,37-39]. This might also influence their perception of privacy regarding any online health information exchange. Furthermore, older patients are highly likely to state “being uncomfortable using online record” as a reason for avoiding online medical records compared to younger patients. The US population had almost 52 million people older than 65 years as of 2018 [40]. This population will be the major consumer of health care systems for the foreseeable future; therefore, any online tools need to be redesigned to be user friendly (ie, for this population to use easily and comfortably).

Finally, our study also showed that patients with cancer use online medical records more than patients who do not have cancer. This was consistent with the findings of previous studies [41-43] showing that patients with such conditions may have a greater need for health tracking and sharing health information with multiple health care professionals than others. Among nonusers of online medical records, patients with cancer are likely to prefer speaking with physicians in person compared to patients who do not have cancer. The complexity of treatment options and the emotional aspect of visits make it more necessary for patients with cancer to meet with doctors in person. On the other hand, the health care system should have alternative plans to maintain quality online visits with these patients during the pandemic.

### Limitations

This study also has limitations. First, the nature of HINTS data is cross-sectional and relies on subjective responses; therefore, it is not able to offer information on causality. Second, the low response rate (20%-30%) might raise some bias concerns, especially related to nonrespondents and sampling strategy. We should also note that the sampling and weighting strategy used by HINTS administrators helps minimize biases and improve national representativeness and generalizability of findings. Nonetheless, some local studies with more detail and a higher response rate should be conducted to validate the findings.

### Conclusion

This study showed factors that lead people to avoid online medical record use across different demographics using a nationally representative survey. The findings show that there is an increased rate of online medical record use compared to previous years; however, this rate is still not at the expected level. The study shows that most patients still prefer speaking in person with their providers instead of using online medical records. Future studies should also look at how the education level of patients impacts these studied factors; our data did not have that component.

We also acknowledge that the recent COVID-19 pandemic has shifted the culture of virtual visits and online medical record use in health care. Future studies should look at online medical record use trends and factors during and after the pandemic to see how these have shifted. Finally, future designs and concepts of online medical communication technologies may also consider the importance of preparing a common ground for patients where different technology acceptance levels are respected.

## Abbreviations

HINTS: Health Information National Trends Survey
